# Uganda's New National Laboratory Sample Transport System: A Successful Model for Improving Access to Diagnostic Services for Early Infant HIV Diagnosis and Other Programs

**DOI:** 10.1371/journal.pone.0078609

**Published:** 2013-11-13

**Authors:** Charles Kiyaga, Hakim Sendagire, Eleanor Joseph, Ian McConnell, Jeff Grosz, Vijay Narayan, Godfrey Esiru, Peter Elyanu, Zainab Akol, Wilford Kirungi, Joshua Musinguzi, Alex Opio

**Affiliations:** 1 Ministry of Health, Kampala, Uganda; 2 College of Health Sciences, Makerere University, Kampala, Uganda; 3 Clinton Foundation Health Access Initiative, Kampala, Uganda; McGill University, Canada

## Abstract

**Introduction:**

Uganda scaled-up Early HIV Infant Diagnosis (EID) when simplified methods for testing of infants using dried blood spots (DBS) were adopted in 2006 and sample transport and management was therefore made feasible in rural settings. Before this time only 35% of the facilities that were providing EID services were reached through the national postal courier system, Posta Uganda. The transportation of samples during this scale-up, therefore, quickly became a challenge and varied from facility to facility as different methods were used to transport the samples. This study evaluates a novel specimen transport network system for EID testing.

**Methods:**

A retrospective study was done in mid-2012 on 19 pilot hubs serving 616 health facilities in Uganda. The effect on sample-result turnaround time (TAT) and the cost of DBS sample transport on 876 sample-results was analyzed.

**Results:**

The HUB network system provided increased access to EID services ranging from 36% to 51%, drastically reduced transportation costs by 62%, reduced turn-around times by 46.9% and by a further 46.2% through introduction of SMS printers.

**Conclusions:**

The HUB model provides a functional, reliable and efficient national referral network against which other health system strengthening initiatives can be built to increase access to critical diagnostic and treatment monitoring services, improve the quality of laboratory and diagnostic services, with reduced turn-around times and improved quality of prevention and treatment programs thereby reducing long-term costs.

## Introduction

Recognizing the urgent need to intensify the global efforts to eliminate HIV and AIDS to achieve an AIDS-free generation, UNAIDS issued the 2011 Political Declaration on HIV and AIDS to significantly scale up efforts to achieve universal access to integrated HIV prevention, treatment, care, and support programs and services to 15 million people [1]. Despite great efforts and growing access to antiretroviral therapy, HIV-related morbidity and mortality remain elevated in many parts of sub-Saharan Africa. In Uganda, the prevalence of HIV infection is estimated at 7% in adults aged 15–49 years and 1.7% in children under 5 years [2]. Never-the-less, early infant diagnosis (EID) of HIV-infected children has helped in the early initiation of antiretroviral therapy, thereby reducing morbidity and mortality [3], [4]. Currently, EID testing in Uganda is done by polymerase chain reaction (PCR) analysis of HIV DNA samples in a centralized laboratory [5] at the central public health laboratories (CPHL) in Kampala.

In 2006, the Uganda EID program adopted simplified dried blood spot (DBS) technology which made testing of infants in rural settings feasible through improved sample management and transport [6]. DBS samples can be transported at room temperature days after initial sample collection, unlike whole blood samples which must be tested on the day of collection [5], [6]. Initially, DBS samples were sent to referral laboratories by different methods and mainly by the Ugandan postal courier, Posta Uganda. The Posta Uganda postal courier system is designed to only reach a 15 km radius outside of their designated collection centers. Most health facilities are outside this 15 km catchment area and therefore could not be reached. The transport of DBS samples from the lower level health units to referral laboratories for HIV DNA PCR was therefore ad-hoc and varied from facility to facility as different methods were used to transport the samples.

In this report we present an innovative specimen transport network system piloted to address the challenges of EID testing and result reporting in Uganda. This system takes into account the challenges of DBS sample referral as well as the cost implications and benefits of a comprehensive sample transport system. We expect this system will be of great value to EID programs in many resource limited settings or even middle income countries of the world and can also impact many other specimen transport systems.

## Methods

### Study design

This was a retrospective study to report on a novel specimen transport network system developed to overcome challenges to EID testing in Uganda. The study population comprised testing of HIV exposed children aged 6 weeks to 18 months, who had Dried Blood Spot (DBS) samples taken for a DNA PCR test between Dec 2006 and Dec 2012, and for whom data on sample collection and test turn-around time were available. HIV-exposed infants were screened and tested for HIV according to the national EID algorithm and national policies.

### Mapping of the HUB transport system

At the initiation of EID services in Dec 2006, samples were transported to 8 testing laboratories across the country by different ad-hoc methods including Posta Uganda. Seven of the 8 laboratories were run by the Joint Clinical Research Center and one was run by MildMay, Uganda. From July 2010 to Nov 2011, EID program consolidated the 8 testing laboratories into one centralized EID laboratory based at the Central Public Health Laboratories (CPHL), run by the Ministry of Health (MoH), in Kampala. The samples were again transported through several ad-hoc means and also by Posta Uganda. In Dec 2010, the Uganda EID program piloted SMS printers at 10 health facilities across the country to improve on result reporting.

From Nov 2011 to Dec 2012, the EID program mapped regional referral hospitals, district hospitals, and other health facilities that could function as sample transport ‘HUBS’, a hub being the coordination center of the sub district network. Using Geographical Information System (GIS) a catchment area of 30 to 40 km radius was mapped around each hub. Using the same GIS, health facilities within that catchment area were identified with respective road networks. Health facilities within that catchment area were identified with respective road networks and bike routes were created so that all health facilities in the catchment could be visited at least once a week. Each hub was provided with a motor bike and a bike rider equipped to perform the daily routes. Each hub served between 20 to 40 health facilities.

**Figure 1:**
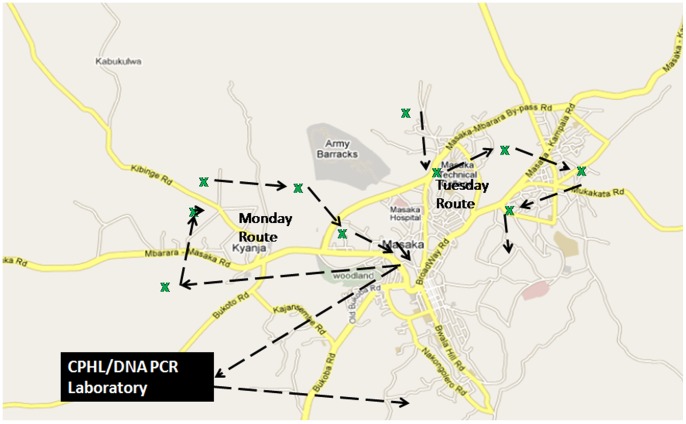
The various methods previously used in transportation of specimen.

Every day, the bike rider embarked on a particular route, visiting between 4 to 8 health facilities. The samples were picked up between 10 am and 4 pm, giving clinicians at health facilities ample time to prepare the samples in the morning. Figure 1 shows the resulting local network that is now covered by each bike within the HUB network system. The schedule created at each HUB allowed the bike rider to reach all the health facilities within the hub catchment areas at least once a week, picking up samples and dropping off results from the previous visit. The following week, the bike rider repeated his/her specific routes, delivering the results from samples dispatched the previous week and picking up new samples.

### Assessment and intervention methods

The new transport system was implemented in a phased manner, which allowed for the first set of hubs to serve as a pilot. The pilot phase has 19 hubs serving 616 health facilities from which a “post-intervention study” was conducted in Dec 2012 by the MoH and Clinton Health Access Initiative (CHAI) to assess the effect on sample-result turnaround time and cost implications. In the pilot, at least 2 study investigators visited each region, where they met with the NGO partner supporting the regions, staff/administrators at the 19 hubs and at least 2 health facilities served by each of the hub (an additional 26 facilities). The investigators discussed the sample-result transport system (s) that were being used in each region, as well as the benefits/drawbacks of the different systems from the perspectives of those involved.

The second part of the investigation was collection of data on 876 sample-result turnaround time (TAT) from 9 selected facilities that cut across different regions and facility levels. Specifically TAT was calculated from the time of specimen collection to the time the result was reported to the patient. The “date of sample collection” was found on the “DBS dispatch form” sent by the facility accompanying the samples, and the “date of result receipt” was found at each individual facility in their DBS logbook.

The third part looked at the costs of DBS sample transport and its relative weight in the overall cost of EID service provision in Uganda. Costs were obtained for each component of the national EID program. Overhead costs for the EID program were accessible at the Ministry of Health; the cost of DBS bundles/reagents/laboratory consumables was obtained at examining the national procurement orders that were submitted, and finally, the cost of sample transport was obtained from the supporting NGOs in each region (their expenditure on sample-result transport). The regional sample transport costs were aggregated, and the relative percentage that each cost category occupied was calculated. Data from a few partners using different models also enabled calculation of the cost per facility for transport of samples and results (i.e. per trip).

### Data Analysis

Data was entered into a Microsoft Excel spreadsheet, cleaned and checked for consistency. The cleaned data was exported to SSPP 10 statistical software for data management and further statistical analysis. The TAT was the major outcome variable in this study and was defined as time for processing the DBS sample, in days, between DBS sample collection and return of DNA PCR results to the facility. Statistical analysis was done on samples before the hubbed system (Dec 2006 to Nov 2011) and after the introduction of the hubbed system (Nov 2011-Dec 2012). All tests were two-sided and statistical significance was set at p value <0.05. From these models, we estimated odds ratios along with 95% confidence intervals for each of the time periods.

## Results

### The HUB specimen transport network system and increased specimen volume

**Figure 2:**
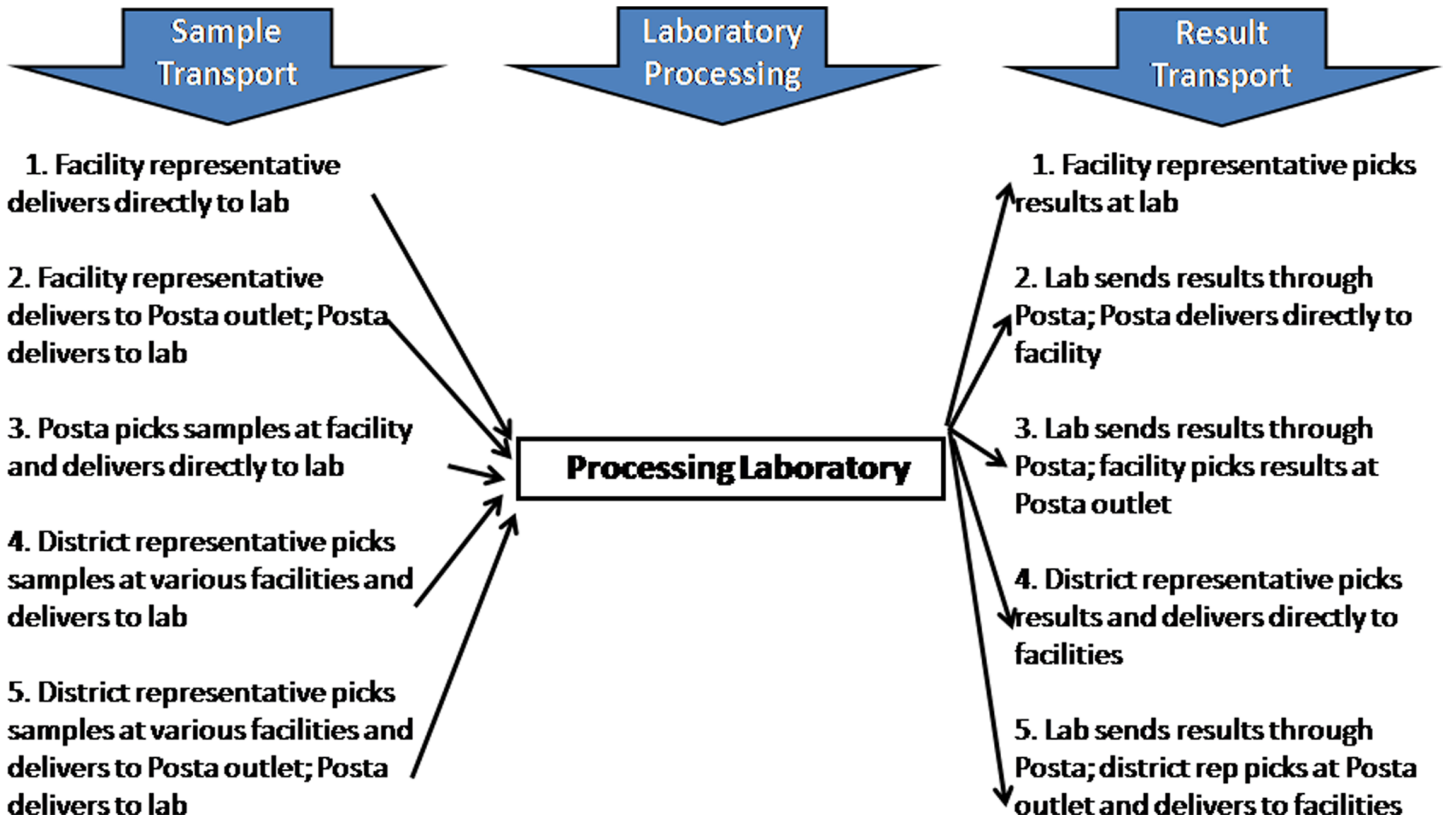
The recommended turnaround time measured as the time from collection of a sample to receipt of the results was about 28

**Figure 3:**
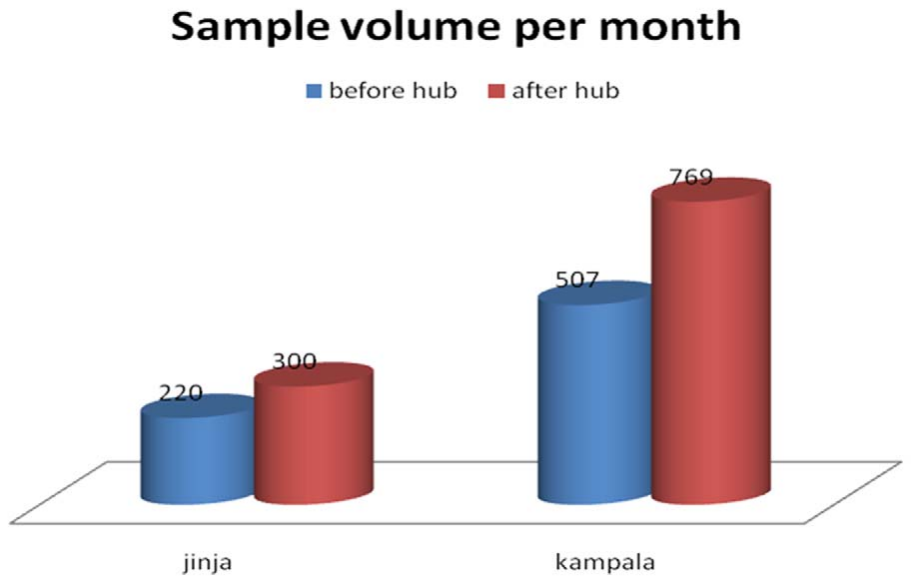
Estimated costs different aspects of sample analysis.

Figure 2 shows the various methods that were used for transport of specimen from 2006 to 2011. During that time only 35% of the facilities that were providing EID services were reached by Posta Uganda. Many health facilities held samples for 3–4 weeks for lack of means to transport them. Following the improvement in the specimen transport system, the HUB network provided increased access to EID testing as showed in Figure 3. With in just one year (Nov 2011- Dec 2012) Jinja, for example, increased EID sample testing volumes by 36.4% from 220 to 300 specimens per month and Kampala increased by 51.7% from 507 to 769 specimens per month.

### Test turn-around time

**Figure 4:**
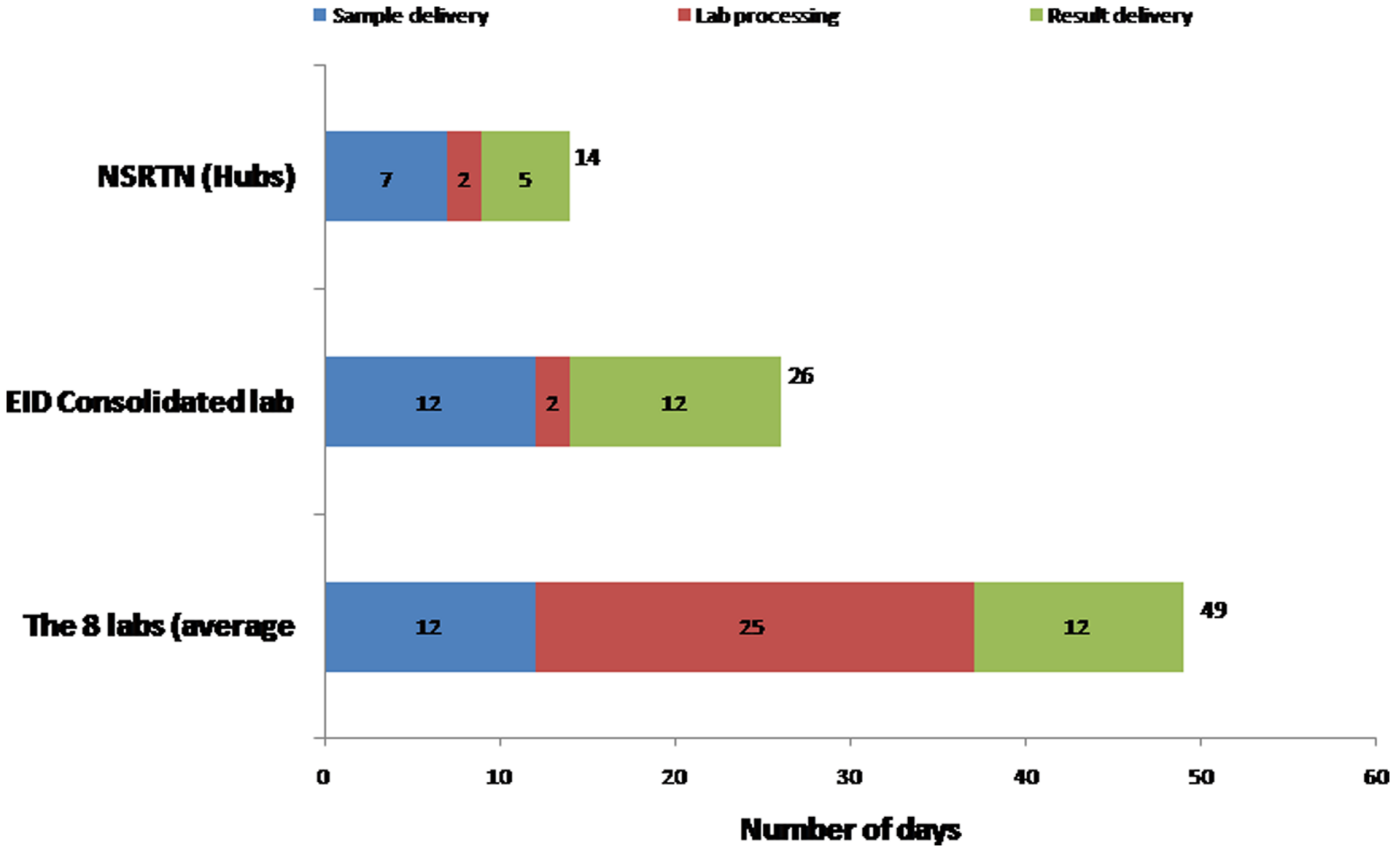
The local network that is covered by each bike.

Test turn-around times were reduced with the HUB transport system. Figure 4 shows how test turn-around times were reduced as a result of consolidating EID services into one central testing facility at CPHL and then further reduced with the introduction of the HUB network system. In 2010, prior to centralized testing at CPHL, average TAT was 49 days. The average TAT for facilities further away from the capital Kampala and for more rural locations (Kayunga Hospital, Bukalula Health Center IV, and Masaka Hospital), was greater than 55 days. After centralization, lab processing was reduced to 2 days, sample delivery decreased from 12 days to 7 days and results delivery decreased from 12 days to 5 days. Overall TAT for all 19 facilities (n = 876) dropped from 49 to 26 days (46.9% reduction and standard deviation 6.85 days). In Dec 2010, the Uganda EID program piloted SMS printers at 10 health facilities across the country to improve on result reporting. TAT decreased again, from 26 days to 14 days (46.2% reduction), suggesting that physically transporting results was a major driver of long turn-around times.

### Cost

**Figure 5:**
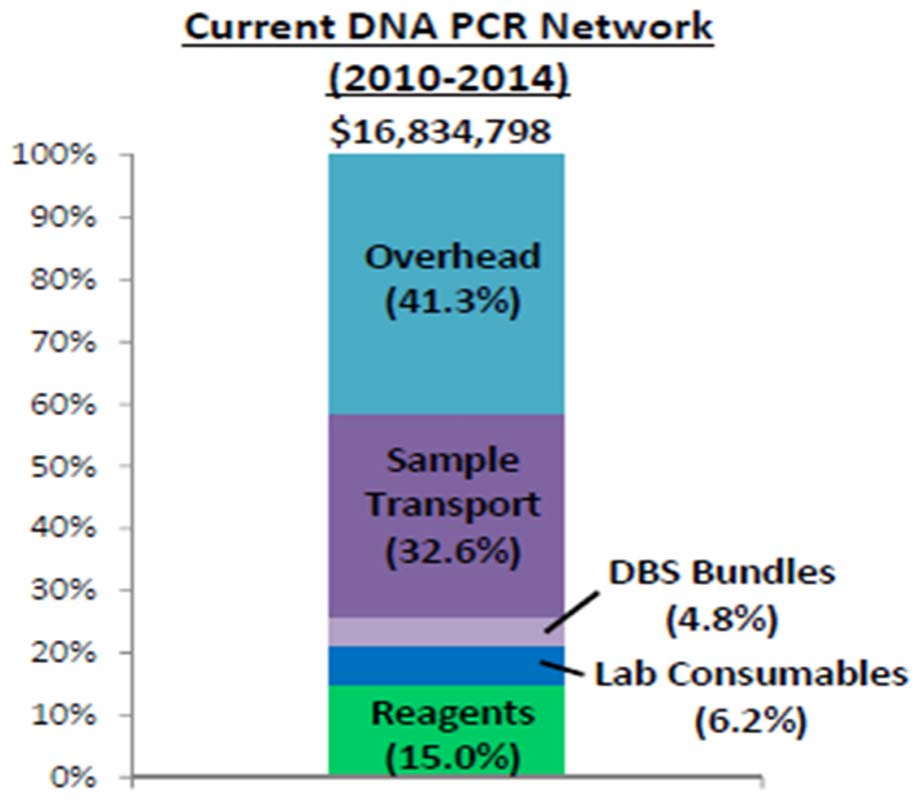
The cost in 2010 before the network was started compared to when additional costs for start up of the transport network were added.

**Figure 6:**
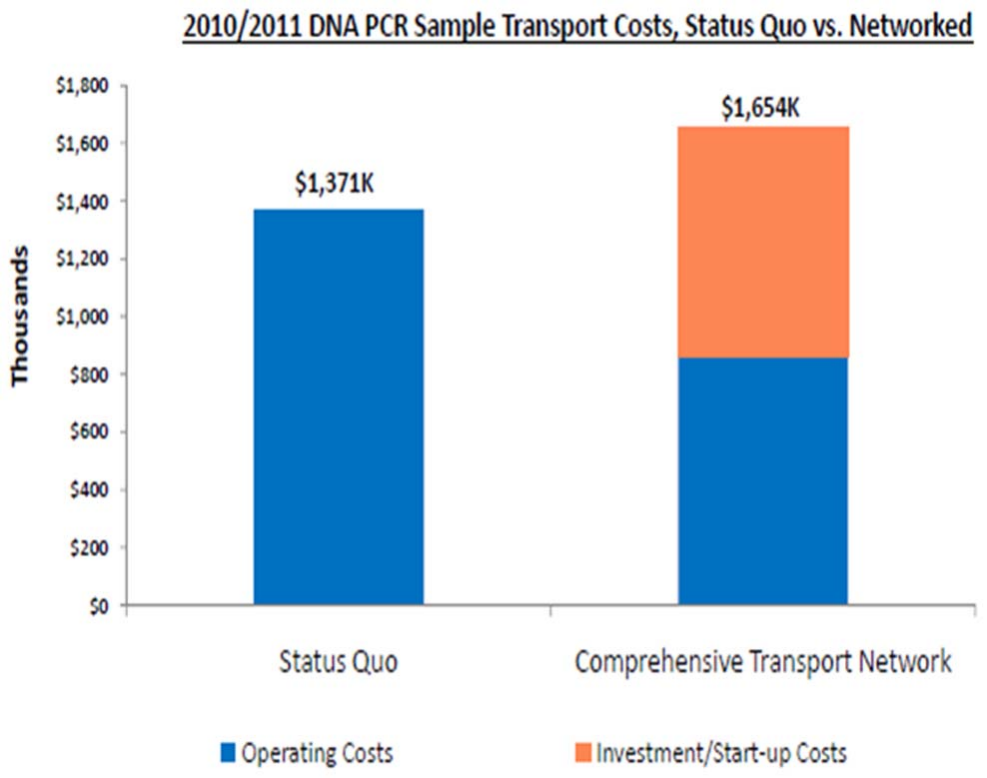
The projected 4 year costs once the transport network is established.

Figure 5 shows the estimated overall cost of USD 16,834,798, for over a period of 4 years, of implementing the National EID program. Out of that, 41.3% was attributed to program overhead costs (i.e. operational costs), 26% to the combination of DBS bundles, laboratory consumables and reagents, and 32.6% to sample transport. Figure 6 shows the country operating costs in 2010, prior to the HUB system, compared to the costs for the comprehensive transport system. Information from 2 NGO partners who transported samples from each facility directly to the regional laboratory revealed that it cost an estimated USD39.71 per trip between the health facility and the regional laboratory. From another NGO partner which provided money to the district laboratory focal person to pick up samples from individual facilities and bring them to the regional laboratory the cost was estimated at USD13.30 per facility per trip.

**Figure 7:**
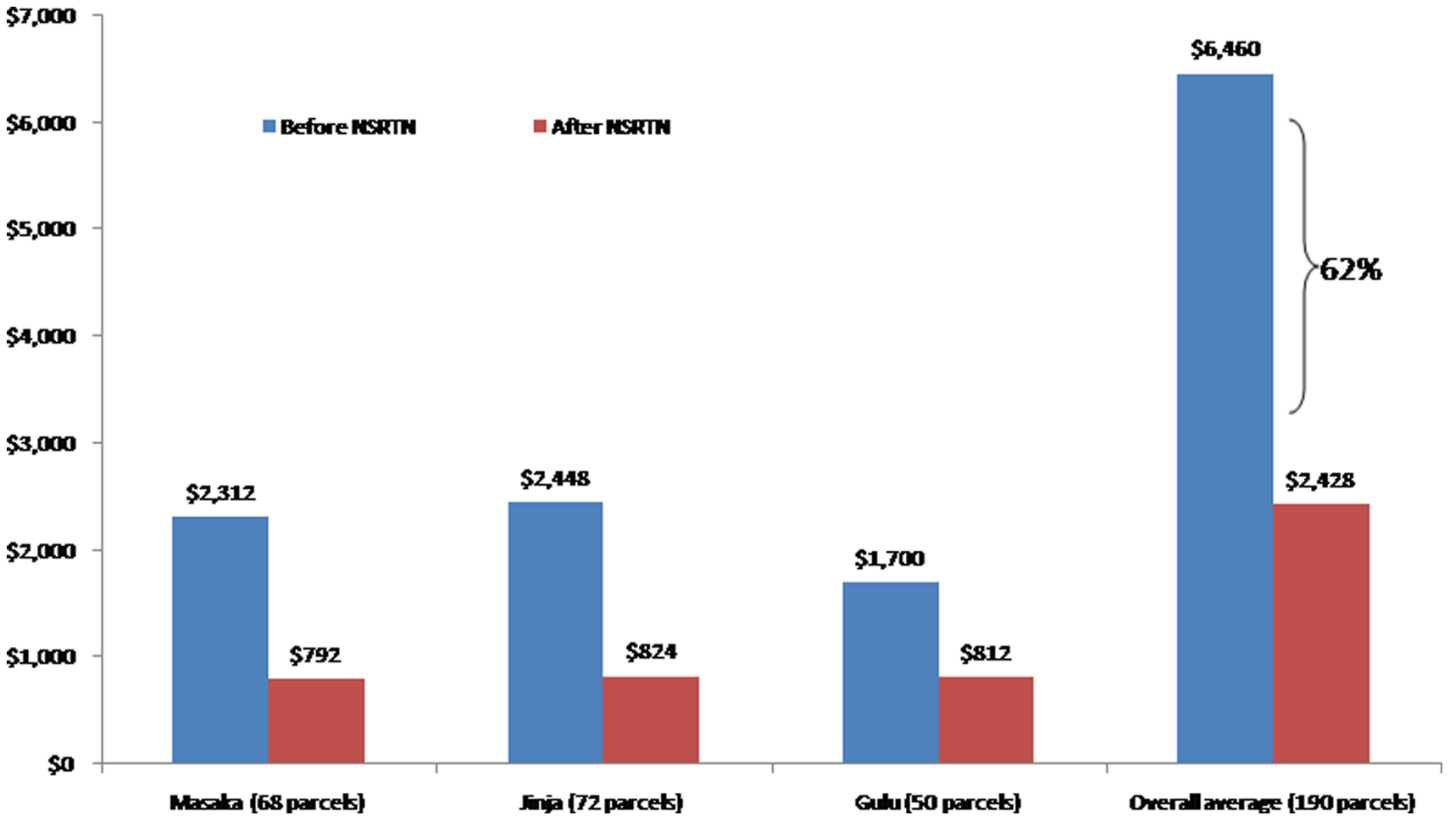
The cost at each of the 19 hubs before and after the initiation of the National sample referral transport network (NSRTN).

Figure 6 shows the increase in overall costs for the sample transport system in the first year of the program (2011) from the old system (2010) by USD 285,000, although a significant proportion was due to the start-up costs (procurement of the bikes, training of hub staff, procurement of protective clothing and specialized sample transport bag, etc). The cost purely due to transport of samples/results, however, would go down substantially. Figure 7 provides a more comprehensive breakdown of costs at 3 of the 19 hubs where pre and post intervention data was available. In these 3 hubs operational costs significantly decreased such that the overall cost for 190 parcels reduced by 62% (from USD 6,460 before intervention to USD 2,428 after the intervention).

**Figure 8:**
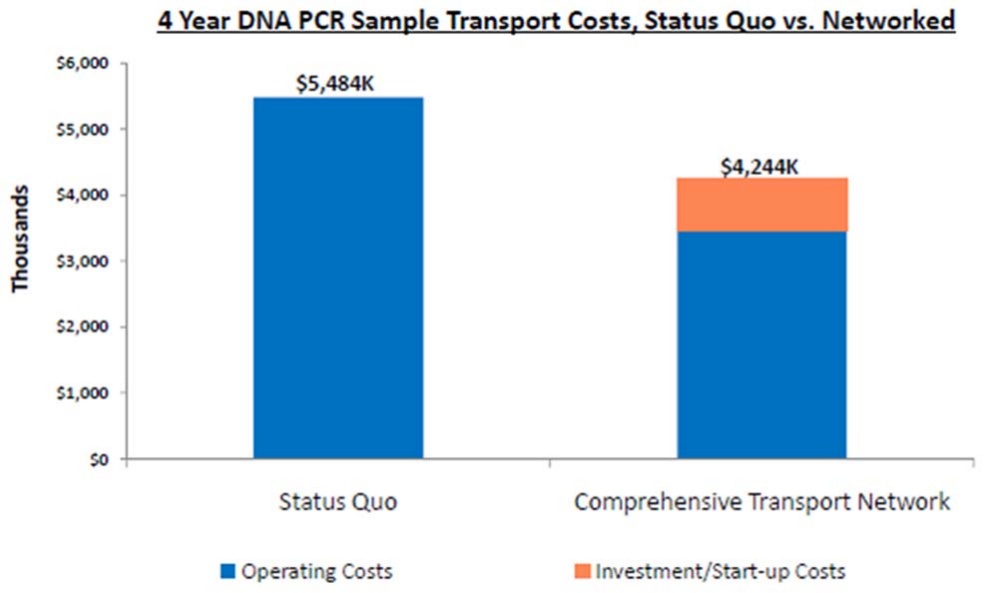
How Turnaround Time reduced; sample and result transit time dropped from 49 days before lab consolidation to 26 day and from 26 to 14 days due to the NSRTN.

Figure 8 also shows what the estimated costs would be over a four-year period if the status quo was maintained (i.e. the old ad hoc system) or if the new sample transport system was used, including the start-up investment costs associated with implementing the new system. The old ad hoc system would have cost USD 5,484,000 over four years, whereas the new system would only cost USD 4,244,000, and USD 1,200,000 could be saved with transportation of DBS samples and results alone.

## Discussions

In Uganda there are approximately 1,700 health facilities at the level of Health Center (HC) III and above, which provide the minimum level of basic laboratory services. Due to health care infrastructure and human resource constraints, higher level laboratory analysis is referred and therefore requires efficient sample transportation to, and result dispatch from, centralized district, regional or national referral laboratories.

At the start of the EID program in 2006, Posta Uganda, our major courier provider, was only able to reach urban areas (which comprise only 35% of the facilities providing EID services) to pick up samples and deliver results. This low cover led to the different methods being used for transport of samples from, and results back to, rural areas. Numerous health facilities kept samples at their facilities for 3–4 weeks because they had no way of transporting them to the nearest main town that had a Posta Uganda pickup outlet. This was one of the reasons for the long TAT. For those facilities that were transporting samples, the supporting NGO partner always paid a lot of money in transport refunds to health workers, but this would not be sustainable in the long run. The supporting NGO for the given region would provide regular financial allowances to facility staff personnel to deliver the samples to the nearest local Posta outlet, from where they would then be sent to the regional reference laboratory. In other cases, a facility staff person would be given a financial allowance by the NGO to deliver samples directly to the regional referral laboratory, by passing the Posta system. Besides being costly, this system negatively impacted on the health care system as the health workers had to abandon patients.

In some districts, implementing partners supported district laboratory focal persons (DLFP) who would pick up samples from all the facilities within the district and deliver them either directly to the regional reference laboratory or to the nearest Posta outlet for mailing. This resulted in unacceptable long TAT as the DLFP would make this route only once or twice a month.

The methods for returning results to the health facility were even more challenging. What made result distribution even more complicated was the absence of communication between the regional reference laboratory, Post Office, district offices, and health facilities. Facility and district staff often did not know that results had arrived at the Posta Outlet or ready for pick up at the regional lab, and consequently the results would sit at the Posta outlet or regional laboratory for weeks. There was also no communication between facilities/districts and the reference laboratory when results were missing. Consequently, these resulted in long TATs.

In 2010, the Ministry of Health centralized EID laboratory testing and scaled-up its EID program to more PMTCT sites. From the analysis, it is seen that expensive yet weak transport systems were ineffective. Extraordinarily large proportion of EID funds were devoted to sample transport, showing that the ad-hoc systems for sample-result transport were costing an excessive amount which was financially unsustainable as the EID program rapidly continued its expansion to more rural facilities.

During the initial scale up of EID services, the Uganda Ministry of Health considered several options. One of them was to identify a third party (Business firm) to hire, train, and coordinate a fleet of motorcycle couriers. The firm would be well-versed on sample transportation practice, and would have specialized backpacks and coolers to facilitate the safe transport of delicate samples. This turned out to be very expensive, the option the MOH took, however, was to set up an organized transport system that would increase access to critical diagnostics beyond EID, improve the quality of laboratory services, and reduce long-term costs. This is the system that was piloted in the 19 hubs.

**Figure 9:**
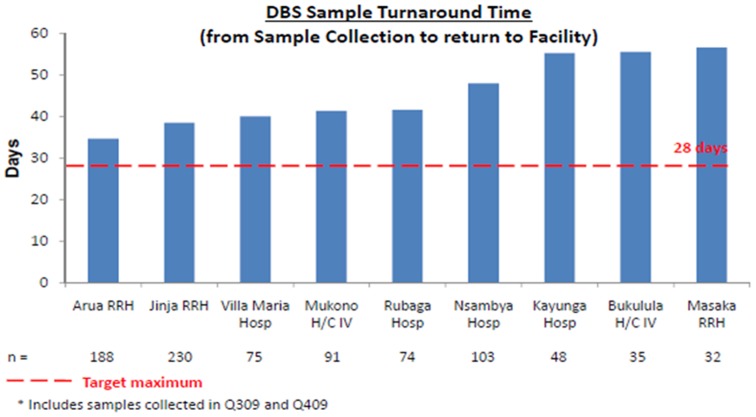
Turnaround times for the facilities before the introduction of the transport network in July 2011. The recommended turnaround time measured as the time from collection of a sample to receipt of the results was about 28

Despite the targeted 28 day maximum turn-around time recommended by the Uganda national guidelines, all sites experienced unacceptably long turn-around times which in some cases went close to 60 days as showed in Figure 9. The major contributor to this long turnaround time was the time sample waited at the facilities before being picked, the time lost in transit and the time lost at the laboratory before analysis. A lot of lost time was associated to the lack of a systematic sample and results transport system.

The EID program handled the issue of time lost at the laboratory by setting up an efficient EID centralized laboratory, which reduced the laboratory turnaround time from 25 days to 2 days as shown by figure 4. The EID program then embarked on the transit time by setting up the sample transport network which reduced sample transport time from 12 days to 7 days and result transit time from 12 days to 5 days as shown by figure 4. The cumulative 49 days turnaround time was reduced to 14 days due to interventions of both laboratory consolidation and the sample transport system.

### Benefits of a Comprehensive Sample Transport System for National Prevention and Treatment Programs

As health service delivery in Uganda continued to decentralize and programs such as EID scaled up, sample transport became increasingly challenging, impacting patient testing and care. The HUB system is being expanded to include other sample types and improve the quality of laboratory services throughout Uganda. This will impact a cross section of other sample types (chemistry, hematology, CD4, sputum, and malaria smears) with similar transportation challenges. Not only does creating a coordinated national transport system free up limited funds for other program areas, it also benefits the existing prevention and treatment programs in Uganda. It is striking to consider how this simple yet effective system could also allow HIV-positive mothers at all HC IIIs facilities and beyond to access CD4 testing; benefiting their own health and preventing HIV transmission to their unborn child.

Sample transportation is a classic economic example where it is much less costly to combine operations through central coordination than it is to support individual facilities by implementing partners and different programs. If the coordinated specimen transport system in a period of 4 years would save USD 1.2 million in transporting EID samples alone, we can infer that even larger amounts are being saved by utilizing the same transport system for CD4 tests, chemistry tests, hematology tests, sputum samples, blood smears, pathology and other samples into this sample transport network. Consolidating many individual ad-hoc operations into one streamlined, centrally coordinated national system has greatly reduced the costs of sample-result transport.

### Projections for the future; Operational and Cost Implications of a Comprehensive HUB Transport System

In a little more than a year, this robust sample transportation system has provided weekly laboratory access through scheduled transportation to every HC III and above in Uganda. Most importantly, this comprehensive network has increased access to laboratory services and reduced turn-around time by providing systematic transport for both samples and results.

The initial investment in the 19 hubs for the year 2011–2012 was USD 248,900 which transported 53003 DBS samples besides other samples. Since bike procurement, training of staff, procurement of protective clothing and specialized sample transport bag, are a onetime investment, costs for subsequent years will reduce.

The next steps are to continue to scale up the hub system to about 100 hubs in the country so that the NSRTS can serve all facilities in Uganda, set up GSM printers at every hub to ease transmission of results from the central laboratory to the hubs and further shorten TAT, strengthen laboratory capacity at the existing transport hubs to perform additional tests (e.g. CD4, CBC and chemistry tests) and thus reduce the number of samples that need to be referred to Kampala, improve integration of different sample types within the hub system, and strengthen day-to-day operation and implementation.

### Conclusion

The HUB system is a functional model against which other health system strengthening initiatives can be built. Despite the challenges, a national sample transport system if scaled up will:

1. Provide a functional, reliable, efficient national referral network that cuts across all sample types
2. Increase access to critical diagnostic and treatment monitoring services
3. Improve the quality of laboratory and diagnostic services, with reduced turn-around times
4. Improve the quality of prevention and treatment programs
5. Reduce long-term costs

The implementation of the national NSRTN for EID services in Uganda has integrated all laboratory services (TB, outbreak investigations, surveillance, histopathology, quality assurance, etc.), it is also strengthening the integration and roll-out of additional HUBS to serve over 1700 new health facilities. In terms of providing high-quality diagnosis and treatment, the benefits of increased access to timely laboratory services are clear – establishing a national transport system as shown in Uganda could transform the quality of prevention and treatment programs and is a worthwhile investment to save both money and lives.
