# Correction: Uganda's New National Laboratory Sample Transport System: A Successful Model for Improving Access to Diagnostic Services for Early Infant HIV Diagnosis and Other Programs

**DOI:** 10.1371/annotation/58b80505-5c73-428a-b8ae-69f7c6f2080c

**Published:** 2014-01-13

**Authors:** Charles Kiyaga, Hakim Sendagire, Eleanor Joseph, Ian McConnell, Jeff Grosz, Vijay Narayan, Godfrey Esiru, Peter Elyanu, Zainab Akol, Wilford Kirungi, Joshua Musinguzi, Alex Opio

The figure legends for this manuscript are in an incorrect order. The correct figure legends are:

Figure Legend 1: Map of the local network that is covered by a bike rider in Masaka, Uganda

Figure Legend 2: The various methods previously used in transportation of specimen

Figure Legend 3: Increase in numbers of samples tested before and after July 2011, the increase attributed to better access to EID testing

Figure Legend 4: Overall turnaround Time reduced; sample and result transit time dropped from 49 days before lab consolidation to 26 day and from 26 to 14 days due to the National Sample Result and Transport Network. 

Figure Legend 5: Estimated costs for different aspects of sample analysis

Figure Legend 6: The cost in 2010 before the network was started compared to 2011 when additional costs for start up of the transport network were added.

Figure Legend 7: The cost of transport for parcels at 3 of the 19 hubs before and after the initiation of the National sample referral transport network (NSRTN) and the difference in total cost of transporting 190 parcels.

Figure Legend 8: The projected 4 year costs (from 2011-2014) once the transport network is fully established.

Figure Legend 9: Turnaround times for the facilities before the introduction of the transport network in July 2011.The recommended turnaround time measured as the time from collection of a sample to receipt of the results was about 28 days.

